# Supplementary material for: A Leucine-Rich Repeat Receptor-like Kinase TaBIR1 Contributes to Wheat Resistance against Puccinia striiformis f. sp. tritici
Source: Int J Mol Sci. 2023 Mar 29;24(7):6438. doi: 10.3390/ijms24076438 (PMC10095076; doi:10.3390/ijms24076438)
Supplement: Supplementary file 1 [file ijms-24-06438-s001.zip › ijms-2307906-supplementary.pdf]

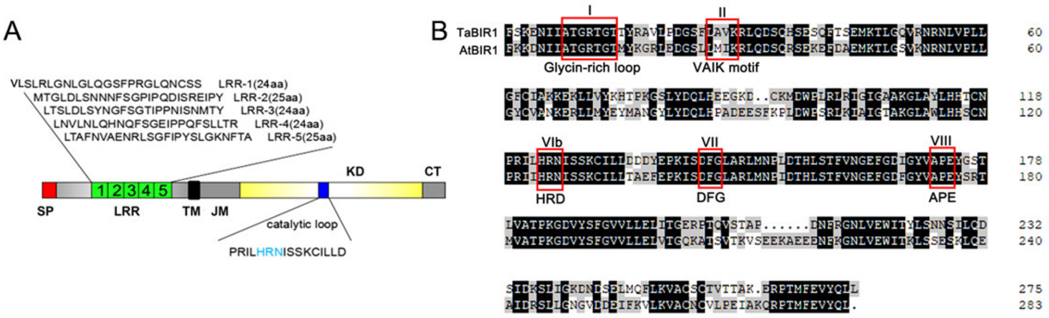

Figure S1. Bioinformatics analysis and sequence alignment for *TaBIR1*. (**A**) Schematic diagram of TaBIR1 protein. SP, signal peptide; LRR, leucine-rich repeat motif; TM, transmembrane domain; JM, juxtamembrane domain; KD, kinase domain; CT, carboxyl-terminal. (**B**) Multiple alignment of intracellular kinase domains of TaBIR1 and its homologue AtBIR1 in Arabidopsis. The conserved subdomains were marked in red boxes.

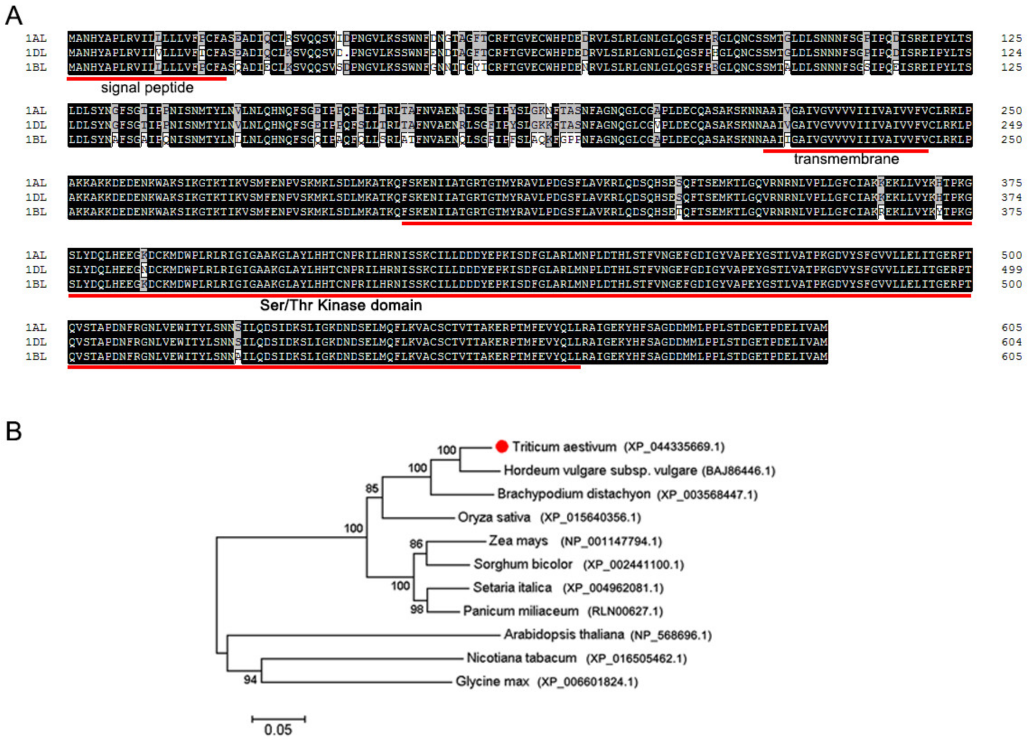

Figure S2. (**A**) Protein sequences alignment of the three homeologues of TaBIR1 from wheat A/B/D subgenomes. The red lines indicated the signal peptide, transmembrane

domain and cytoplasmic serine/threonine kinase domain of TaBIR1. (**B**) Phylogenetic relationship of TaBIR1 and the homologous proteins. The tree was carried out with the MEGA7 software by the neighbour-joining method.

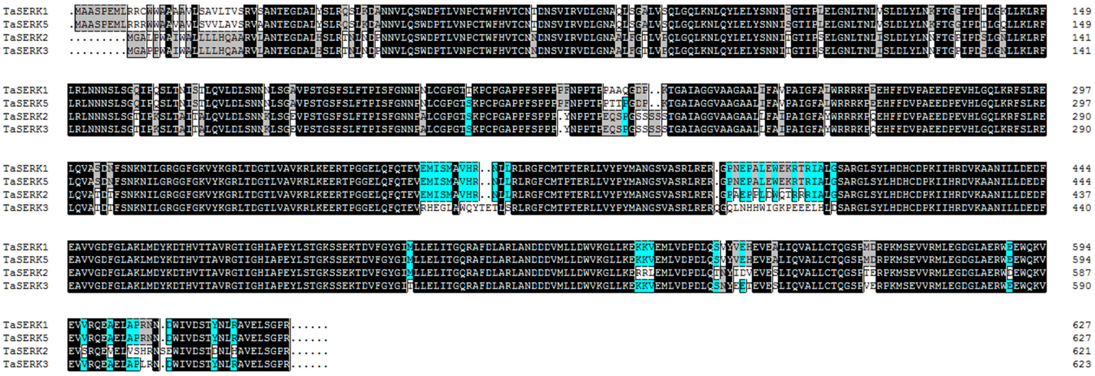

Figure S3. Sequences alignment of four wheat TaSERKs. The reference sequences were retrieved from Accession no. AK333001(*TaSERK1*); Accession no. AK333677.1(*TaSERK2*) ; Accession no. BT009223(*TaSERK3*); Accession no. BT009426 (*TaSERK5*) in NCBI database followed by a cDNA clone in wheat variety Su11. The protein sequences alignment were achieved by DNAMAN6.0 software.

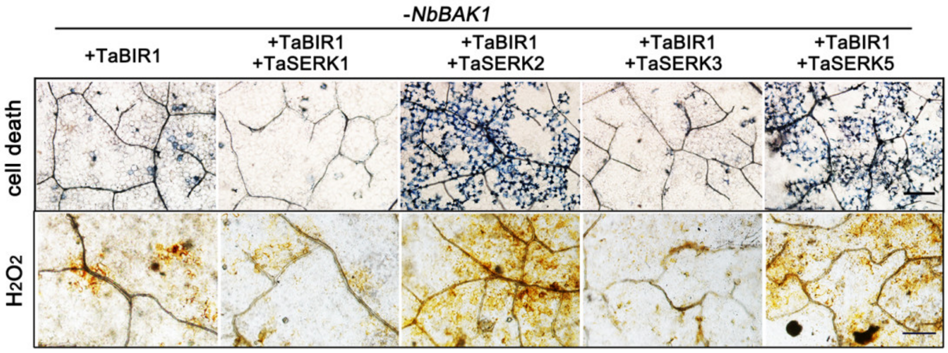

Figure S4. ROS accumulation and cell death in *NbBAK1* silencing plant leaves coexpressing TaBIR1 and TaSERKs. Two weeks after inoculation of TRV2:*NbBAK1*, *TaSERKs* and *TaBIR1* were co-expressed in the corresponding plant leaves. Forty-eight hours later, the leaves were collected for trypan blue staining and DAB staining for observations of cell death and H_2O_2. Similar results were obtained in three independent experiments. Bars, 100 μm.

Table S1. Primers used in this study.

**Primer name**	**Sequence(5'-3')**	**Purpose**
TaBIR1-qRT-F	GTAATCAAGGGCTATGTGG	qRT-PCR analysis in

TaBIR1-qRT-R	TCCTTTTTTGCTTTCTTGGC	wheat (also for VIGS efficiency)
TaBIR1(VIGS-1)-γ-F	TTAGCTAGCTGATTAATTAATCAGCAGTCAGTGATTGACCC	vectors construction for wheat VIGS
TaBIR1(VIGS-1)-γ-R	TCCGTTGCTAGCTGAGCGGCCGCCTACAATTCTGAAGACCGCGA	
TaBIR1(VIGS-2)-γ-F	TTAGCTAGCTGATTAATTAAATCATTTCTCGGCCGGGGACG	
TaBIR1(VIGS-2)-γ-R	TCCGTTGCTAGCTGAGCGGCCGCTGCAGCGTACCGCTACTCCAC	
GFP-VIGS-γ-F	TTAGCTAGCTGATTAATTAAATGATATAGACGTTGTGGCTGTTGT	
GFP-VIGS-γ-R	TCCGTTGCTAGCTGAGCGGCCGCAGCACGACTTCTTCAAGTCCGCCAT	
qRT-TaEF-F	TGGTGTCATCAAGCCTGGTATGGT	qRT-PCR analysis of PTI genes in wheat VIGS
qRT-TaEF-R	ACTCATGGTGCATCTCAACGGACT	
qRT-PsEF-F	TTCGCCGTCCGTGATATGAGACAA	
qRT-PsEF-R	ATGCGTATCATGGTGGTGGAGTGA	
qRT-TaPR1-F	AAACAACATCCATCAAAACCA	
qRT-TaPR1-R	ATTACCAGGTGGATCATAGTTACA	
qRT-TaPR2-F	AGGATGTTGCTTCCATGTTTGCCG	
qRT-TaPR2-R	AAGTAGATGCGCATGCCGTTGATG	
Biomass-PstEF-F	TTCGCCGTCCGTGATATGAGACAA	Biomass detection in wheat VIGS
Biomass-PstEF-R	ATGCGTATCATGGTGGTGGAGTGA	
Biomass-WheatEF-F	TGACCAGATCAACGAGCC	
Biomass-WheatEF-R	CTCCAGGAGAGACTCATG	
TaBIR1full-16318GFP	AGAGGACAGCCCAAGCTTATGGCAAATCATTATGCTCCT	vector construction for subcellular

-F		localization in wheat
TaBIR1full-16318GFP-R	GCCCTTGCTCACCATGGATCCCATGGCGACAATGAGCTC	
TaBIR1full-pCAMBIA1302GFP-F	CATGGTAGATCTGACTAGTATGGCAAATCATTATGCTCCT	vector construction for subcellular localization in *N. benthamiana*
TaBIR1full-pCAMBIA1302GFP-R	GCCCTTGCTCACCATCCTAGGCATGGCGACAATGAGCT	
TaBIR1-GFP-F	CTGTACAAGGGTACCCCCGGGATGGCAAATCATTATGCTCCT	vectors construction for TaBIR1 overexpression in *N. benthamiana*
TaBIR1-GFP-R	TCTAGTTCATCTAGAGGATCCTTACATGGCGACAATGAGCT	
qRT-NbActin-F	TGGTCGTACCACCGGTATTGTGTT	qRT-PCR analysis of PTI gene in *N. benthamiana*
qRT-NbActin-R	TCACTTGCCCATCAGGAAGCTCAT	
qRT-NbPR1-F	CCGCCTTCCCTCAACTCAAC	
qRT-NbPR1-R	GCACAACCAAGACGTACTGAG	
qRT-NbPR2-F	AGGTGTTTGCTATGGAATGC	
qRT-NbPR2-R	TCTGTACCCACCATCTTGC	
TaBIR1full-(LUC-N)-F	AACACGGGGGACGAGCTCGGTACCATGGCAAATCATTATGCTCCT	vectors construction for interaction of luciferase complementation assay
TaBIR1full-(LUC-N)-R	TCCTTGTAGTCCATTTGTTGGATCCCGCATGGCGACAATGAGCTC	
TaBIR1full-(LUC-C)-F	AGAGAACACGGGGGACGAGCTCATGGCAAATCATTATGCTCCT	
TaBIR1full	GTTTACATAACCGGACATGAGCTCCATGGCGACA	

-(LUC-C)-R	ATGAGCTC	
TaSERK1-(LUC-N)-F	AACACGGGGGACGAGCTCGGTACCATGGCTGCGTCGCCGGAGATG	
TaSERK1-(LUC-N)-R	TCCTTGTAGTCCATTTGTTGGATCCCCTCGGGCCGGACAGCT	
TaSERK2-(LUC-N)-F	AACACGGGGGACGAGCTCGGTACCATGGGGGCGCTGCCGTGGG	
TaSERK2-(LUC-N)-R	TCCTTGTAGTCCATTTGTTGGATCCCCTTGGCCCTGATAGCT	
TaSERK3-(LUC-N)-F	AACACGGGGGACGAGCTCGGTACCATGGGGGCGCCGCCGT	
TaSERK3-(LUC-N)-R	TCCTTGTAGTCCATTTGTTGGATCCTCTTGGGCCAGACAGTT	
TaSERK5-(LUC-N)-F	AACACGGGGGACGAGCTCGGTACCATGGCTGCGTCGCCGGAGA	
TaSERK5-(LUC-N)-R	TCCTTGTAGTCCATTTGTTGGATCCCCTCGGGCCGGACAGCT	
TaSERK1-ICD(FLAG-HA)-PICH-F	TACAATTATCGATACAATGGATTACAAGGATGACGACGATAAGCGGCGACGTAAACCTG	vectors construction for Co-IP interaction
TaSERK1-ICD(FLAG-HA)-PICH-R	TAAAGCAGGACAAGCttaAGCGTAGTCTGGGACGTCGTATGGGTACCTCGGGCCGGACA	
TaSERK2-ICD(FLAG-HA)-PICH-F	TACAATTATCGATACAATGGATTACAAGGATGACGACGATAAGCGCCGCAGGAAACCGC	
TaSERK2-ICD(FLAG-HA)-PICH-R	TAAAGCAGGACAAGCttaAGCGTAGTCTGGGACGTCGTATGGGTACCTTGGCCCTGATA	
TaSERK3-ICD(FLA	TACAATTATCGATACAATGGATTACAAGGATGACGACGATAAGCGCCGCAGGAAACCGC	

G-HA)-PICH-F		
TaSERK3-ICD(FLAG-HA)-PICH-R	TAAAGCAGGACAAGCttaAGCGTAGTCTGGGACGTCGTATGGGTATCTTGGGCCAGACA	
TaSERK5-ICD(FLAG-HA)-PICH-F	TACAATTATCGATACAATGGATTACAAGGATGACGACGATAAGCGGCGACGTAAACCTG	
TaSERK5-ICD(FLAG-HA)-PICH-R	TAAAGCAGGACAAGCttaAGCGTAGTCTGGGACGTCGTATGGGTACCTCGGGCCGGACA	
TaBIR1-ICD-pBinGFP-F	CTGTACAAGGGTACCCCCGGGATGTGTCTGCGGAAATTACCAGC	
TaBIR1-ICD-pBinGFP-R	TCTAGTTCATCTAGAGGATCCTTACATGGCGACAATGAGCTC	
TaBIR1full-PICH-HA-F	TTACAATTATCGATACAATGTACCCATACGACGTCCCAGACTACGCTATGGCAAATCATTATGCTCCT	HA-tagged vector construction for TaBIR1 expression of VIGS in *N. benthamiana*
TaBIR1full-PICH-HA-R	CTCATTAAAGCAGGACAAGCTTACATGGCGACAATGAGCT	
qRT-NbActin-F	TGGTCGTACCACCGGTATTGTGTT	qRT-PCR analysis of VIGS in *N. benthamiana*
qRT-NbActin-R	TCACTTGCCCATCAGGAAGCTCAT	
qRT-NbBAK1-F	GAGGTGGGAGGAATGGCAAA	
qRT-NbBAK1-R	TTGGCCCCGACAATTCATCT	
qRT-NbSOBIR1-F	CCAGCAAGTCACAGAAGGGA	
qRT-	CCAACACCACACCAAAGCTG	

NbSOBIR 1-R		
